# Carboplatin Induction Chemotherapy in Clinically Lymph Node–positive Bladder Cancer

**DOI:** 10.1016/j.euros.2023.02.014

**Published:** 2023-03-25

**Authors:** Markus von Deimling, Laura S. Mertens, Bas W.G. van Rhijn, Yair Lotan, Philippe E. Spiess, Siamak Daneshmand, Peter C. Black, Maximilian Pallauf, David D'Andrea, Marco Moschini, Francesco Soria, Francesco Del Giudice, Luca Afferi, Ekaterina Laukhtina, Takafumi Yanagisawa, Tatsushi Kawada, Jeremy Y.-C. Teoh, Mohammad Abufaraj, Guillaume Ploussard, Mathieu Roumiguié, Pierre I. Karakiewicz, Marko Babjuk, Paolo Gontero, Evanguelos Xylinas, Michael Rink, Shahrokh F. Shariat, Benjamin Pradere

**Affiliations:** a Department of Urology, Comprehensive Cancer Center, Medical University of Vienna, Vienna, Austria; b Department of Urology, University Medical Center Hamburg-Eppendorf, Hamburg, Germany; c Department of Urology, The Netherlands Cancer Institute, Amsterdam, The Netherlands; d Department of Urology, Caritas St. Josef Medical Centre, University of Regensburg, Regensburg, Germany; e Department of Urology, University of Texas Southwestern, Dallas, TX, USA; f Department of Genitourinary Oncology, H. Lee Moffitt Cancer Center and Research Institute, Tampa, FL, USA; g USC/Norris Comprehensive Cancer Center, Institute of Urology, University of Southern California, Los Angeles, CA, USA; h Vancouver Prostate Centre, Department of Urologic Sciences, University of British Columbia, Vancouver, Canada; i Department of Urology, University Hospital Salzburg, Paracelsus Medical University, Salzburg, Austria; j Department of Urology, Urological Research Institute, Vita-Salute San Raffaele, Milan, Italy; k Division of Urology, Department of Surgical Sciences, San Giovanni Battista Hospital, University of Studies of Torino, Turin, Italy; l Department of Maternal Infant and Urologic Sciences, Sapienza University of Rome, Policlinico Umberto I Hospital, Rome, Italy; m Department of Urology, Luzerner Kantonsspital, Luzern, Switzerland; n Institute for Urology and Reproductive Health, Sechenov University, Moscow, Russia; o Department of Urology, Jikei University School of Medicine, Tokyo, Japan; p Department of Urology, Okayama University Graduate School of Medicine, Dentistry and Pharmaceutical Sciences, Okayama, Japan; q S.H. Ho Urology Centre, Department of Surgery, The Chinese University of Hong Kong, Hong Kong, China; r Division of Urology, Department of Special Surgery, The University of Jordan, Amman, Jordan; s Department of Urology, La Croix Du Sud Hospital, Quint-Fonsegrives, France; t Department of Urology, CHU Toulouse-IUCT Oncopole, Toulouse, France; u Cancer Prognostics and Health Outcomes Unit, Division of Urology, University of Montreal Health Center, Montreal, Canada; v Department of Urology, Hospital Motol, Second Faculty of Medicine, Charles University, Prague, Czechia; w Division of Urology, Molinette Hospital, University of Studies of Torino, Torino, Italy; x Department of Urology, Bichat-Claude Bernard Hospital, AP-HP, Paris University, Paris, France; y Hourani Center for Applied Scientific Research, Al-Ahliyya Amman University, Amman, Jordan; z Karl Landsteiner Institute of Urology and Andrology, Vienna, Austria; aa Department of Urology, Weill Cornell Medical College, New York, NY, USA; bb Department of Urology, Second Faculty of Medicine, Charles University, Prague, Czechia

**Keywords:** Carboplatin, Induction chemotherapy, Oligometastatic, Survival, Urinary bladder neoplasms

## Abstract

**Background:**

There are currently no guideline recommendations regarding the treatment of cisplatin-ineligible, clinically lymph node–positive (cN+) bladder cancer (BCa).

**Objective:**

To investigate the oncological efficacy of gemcitabine/carboplatin induction chemotherapy (IC) in comparison to cisplatin-based regimens in cN+ BCa.

**Design, setting, and participants:**

This was an observational study of 369 patients with cT2–4 N1–3 M0 BCa.

**Intervention:**

IC followed by consolidative radical cystectomy (RC).

**Outcome measurements and statistical analysis:**

The primary endpoints were the pathological objective response (pOR; ypT0/Ta/Tis/T1 N0) rate and the pathological complete response (pCR; ypT0N0) rate. We applied 3:1 propensity score matching (PSM) to reduce selection bias. Overall survival (OS) and cancer-specific survival (CSS) were compared across groups using the Kaplan-Meier method. Associations between the treatment regimen and survival endpoints were tested in multivariable Cox regression analyses.

**Results and limitations:**

After PSM, a cohort of 216 patients was available for analysis, of whom 162 received cisplatin-based IC and 54 gemcitabine/carboplatin IC. At RC, 54 patients (25%) had a pOR and 36 (17%) had a pCR. The 2-yr CSS was 59.8% (95% confidence interval [CI] 51.9–69%) for patients who received cisplatin-based IC versus 38.8% (95% CI 26–57.9%) for those who received gemcitabine/carboplatin. For the pOR (*p* = 0.8), ypN0 status at RC (*p* = 0.5), and cN1 BCa subgroups (*p* = 0.7), there was no difference in CSS between cisplatin-based IC and gemcitabine/carboplatin. In the cN1 subgroup, treatment with gemcitabine/carboplatin was not associated with shorter OS (*p* = 0.2) or CSS (*p* = 0.1) on multivariable Cox regression analysis.

**Conclusions:**

Cisplatin-based IC seems to be superior to gemcitabine/carboplatin and should be the standard for cisplatin-eligible patients with cN+ BCa. Gemcitabine/carboplatin may be an alternative treatment for selected cisplatin-ineligible patients with cN+ BCa. In particular, selected cisplatin-ineligible patients with cN1 disease may benefit from gemcitabine/carboplatin IC.

**Patient summary:**

In this multicenter study, we found that selected patients with bladder cancer and clinical evidence of lymph node metastasis who cannot receive standard cisplatin-based chemotherapy before surgery to remove their bladder may benefit from chemotherapy with gemcitabine/carboplatin. Patients with a single lymph node metastasis may benefit the most.

## Take Home Message

No guideline recommendations exist for the treatment of cisplatin-ineligible patients with clinically lymph node–positive (cN+) bladder cancer. In this study, carboplatin-based induction chemotherapy appeared to be an attractive alternative before radical cystectomy for this population. In particular, patients with cN1 disease who are not eligible for cisplatin may benefit from gemcitabine/carboplatin induction chemotherapy.

## 1 Introduction

Bladder cancer (BCa) accounted for more than 80 000 new cancer cases in the USA in 2021 [1]. Among patients with muscle-invasive bladder cancer (MIBC), up to one-third present with clinically lymph node–positive (cN+) disease on modern staging; these patients have a worse prognosis than for those with clinically lymph node–negative disease [2], [3], [4], [5], [6], [7]. The optimal treatment for cN+ disease has not been established, but most patients seem to benefit from systemic therapy followed by radical cystectomy (RC) in cases with a response to systemic therapy. This population has been excluded from neoadjuvant chemotherapy trials, but according to more modern imaging, some of the patients in the neoadjuvant chemotherapy clinical trials would be today considered as having cN+ status [8], [9], [10].

Currently, eligible patients with cN+ disease receive upfront cisplatin-based combination induction chemotherapy (IC), followed by RC in cases with a clinical response, with some evidence regarding the benefit of consolidative therapy [11], [12], [13], [14]. Nevertheless, despite the benefit of cisplatin-based IC before RC in this setting, little evidence exists regarding the benefit of other IC regimens, specifically for cisplatin-ineligible patients. However, up to 50% of patients with non–organ-confined BCa may be ineligible for cisplatin-based chemotherapy because of renal impairment, hearing loss, other medical comorbidities, and/or poor performance status [15]. Current guidelines provide no conceptual framework for cisplatin-ineligible patients with cN+ disease that is potentially curable.

By analogy to metastatic BCa, for which carboplatin is recommended for cisplatin-ineligible patients and achieves response rates up to 44% [16], [17], gemcitabine/carboplatin IC is often considered for patients with cN+ disease. The aim of this study was to compare the oncological efficacy of gemcitabine/carboplatin IC versus cisplatin-based combination IC in patients with cN+ BCa. Both groups underwent RC as consolidative therapy. Pathological and survival endpoints were compared between the groups.

## 2 Patients and methods

### 2.1 Study population

This is a retrospective fixed cohort study. We retrospectively reviewed an established multi-institutional database of patients with cN+ BCa treated with IC followed by consolidative RC with bilateral pelvic lymph node dissection (PLND) between 1999 and 2021. We included patients with cT2–4 N1–3 M0 disease at staging who received IC before RC. IC was defined as preoperative receipt of at least two cycles of multiagent chemotherapy that had to have been followed within 6 mo by RC. Patients were stratified according to their initial chemotherapy regimen as a cisplatin-based or gemcitabine/carboplatin regimen. Patients who received other systemic therapy regimens, patients with missing or unknown data for clinical or pathological TNM status or survival outcomes (overall survival [OS], cancer-specific survival [CSS]), and patients treated with RC alone or for reasons other than BCa were excluded. Data collection was approved by the local ethics committee at all participating institutions and informed consent for participation in retrospective studies was obtained where necessary.

Clinical lymph node status was determined via routine imaging during the preoperative workup using a computed tomography scan or magnetic resonance imaging of the pelvis and abdomen. Pelvic lymph nodes were considered metastatic when their maximum short axis exceeded 8 mm [18]. Images were not reassessed. At RC, the extent of PLND was at the surgeon’s discretion. Standard PLND included the internal and external iliac and obturator lymph nodes. Extended PLND also included the common iliac and presacral lymph nodes. Finally, super-extended PLND included all pelvic and abdominal lymph nodes up to the root of the inferior mesenteric artery. Clinical and histopathological stages were classified according to the most recent American Joint Committee on Cancer TNM staging system. Each surgical specimen was histologically examined by an experienced, dedicated pathologist at each center. There was no central pathology review.

Follow-up was performed according to contemporary guideline recommendations and included clinical assessment, diagnostic imaging of the abdomen and pelvis, including the urinary tract, and chest radiography.

The primary endpoint of our study was the pathological response to chemotherapy, defined as a pathological objective response (pOR; ypT0/Ta/Tis/T1 N0) or a pathological complete response (pCR; ypT0 N0). We considered any tumor or nodal downstaging in patients not classified as having a pOR or pCR as any response (pAR), and all other cases as no response (pNR). The secondary endpoints were ypN status at RC, OS, and CSS. We defined the follow-up duration as the time from RC until last follow-up or death. Patients were censored at their last follow-up.

### 2.2 Propensity score matching

We used propensity score matching (PSM) to adjust for different sample sizes and to balance the characteristics of different treatment groups. We performed 3:1 PSM according to the nearest neighbor using logistic regression. We used age, sex, presence of carcinoma in situ at transurethral resection of bladder tumor, clinical tumor stage, and clinical node stage for balancing across treatment groups. The standardized mean difference (SMD) was assessed for quality control of the matching (SMD ≤0.1; Supplementary Fig. 1).

### 2.3 Statistical analysis

Results are reported as the frequency and proportion for categorical variables and as the mean and standard deviation (SD) for continuous variables. Data for continuous variables were tested for a normal distribution using the Shapiro-Wilk test. If normally distributed, continuous variables were compared using a two-sample independent *t* test. Continuous variables with a non-normal distribution were compared using the Wilcoxon rank-sum test. All categorical variables were compared using a χ^2^ test or Fisher’s exact test, as appropriate.

We used logistic regression modeling to test the association of the chemotherapy regimen administered with pCR, pOR, pNR, and ypN status at RC. Survival differences between groups were evaluated using the Kaplan-Meier method and significance across groups was tested with the log-rank test. Multivariable Cox proportional-hazards models were applied to assess the association of clinicopathological features with OS and CSS in the overall cohort as well as in cN subgroups.

Statistical analysis was performed using R v4.1.2 (R Foundation for Statistical Computing, Vienna, Austria). All tests were two-sided, and *p* values <0.05 were considered statistically significant.

## 3 Results

### 3.1 Baseline characteristics

In total, 369 patients fulfilled the inclusion criteria, of whom 315 received cisplatin-based IC and 54 gemcitabine/carboplatin (Supplementary Table 1). After 3:1 PSM, 216 patients were eligible for further analysis. Table 1 lists clinicopathological features for these 216 patients. After PSM, there were no significant differences between the IC regimen groups. In the cisplatin group, 104 patients had received gemcitabine/cisplatin and 58 had received methotrexate, vinblastine, doxorubicin, and cisplatin (MVAC; 22 dose-dense MVAC and 36 MVAC). The clinical nodal status distribution was 54% cN1, 40% cN2, and 6.2% cN3 in the cisplatin group, and 50% cN1, 43% cN2, and 7.4% cN3 in the carboplatin group.

**Table 1:** Baseline characteristics of a propensity score–matched cohort of 216 patients treated with induction chemotherapy and radical cystectomy with LND for cT2–4 N1–3 M0 bladder cancer a

Parameter	Overall	Regimen		
	Cohort (*N* = 216)	Cisplatin (*N* = 162)	Carboplatin (*N* = 54)	*p* value b
Mean age, yr (SD)	65.7 (8.4)	65.6 (8.0)	66.2 (9.8)	0.8
Sex, *n*/*N* (%)				0.6
Female	51/216 (24)	37/162 (23)	14/54 (26)	
Male	165/216 (76)	125/162 (77)	40/54 (74)	
Smoking history, *n*/*N* (%)				0.4
Current smoker	40/170 (24)	34/133 (26)	6/37 (16)	
Never smoker	64/170 (38)	47/133 (35)	17/37 (46)	
Past smoker	66/170 (39)	52/133 (39)	14/37 (38)	
CIS at TURBT, *n*/*N* (%)	15/216 (6.9)	11/162 (6.8)	4/54 (7.4)	>0.9
Clinical T stage, *n*/*N* (%)				>0.9
cT2	94/216 (44)	71/162 (44)	23/54 (43)	
cT3	66/216 (31)	49/162 (30)	17/54 (31)	
cT4	56/216 (26)	42/162 (26)	14/54 (26)	
Clinical N stage, *n*/*N* (%)				0.8
cN1	114/216 (53)	87/162 (54)	27/54 (50)	
cN2	88/216 (41)	65/162 (40)	23/54 (43)	
cN3	14/216 (6.5)	10/162 (6.2)	4/54 (7.4)	
Variant histology at TURBT	21/216 (9.7)	16/162 (9.9)	5/54 (9.3)	0.9
Number of chemotherapy cycles, *n*/*N* (%)				0.5
≤3 cycles	55/216 (25)	39/162 (24)	16/54 (30)	
4 cycles	114/216 (53)	89/162 (55)	25/54 (46)	
≥5 cycles	47/216 (22)	34/162 (21)	13/54 (24)	
Pathological T stage, *n*/*N* (%)				0.4
ypT0	56/216 (26)	43/162 (27)	13/54 (24)	
ypTa/pTis/pT1	24/216 (11)	16/162 (9.9)	8/54 (15)	
ypT2	26/216 (12)	23/162 (14)	3/54 (5.6)	
ypT3	67/216 (31)	50/162 (31)	17/54 (31)	
ypT4	43/216 (20)	30/162 (19)	13/54 (24)	
Pathological N stage, *n*/*N* (%)				0.5
ypN0	102/216 (47)	77/162 (48)	25/54 (46)	
ypN1	41/216 (19)	29/162 (18)	12/54 (22)	
ypN2	53/216 (25)	43/162 (27)	10/54 (19)	
ypN3	20/216 (9.3)	13/162 (8.0)	7/54 (13)	
Pathological N status, *n*/*N* (%)				0.9
ypN+	114/216 (53)	85/162 (52)	29/54 (54)	
Objective response (pOR)	54/216 (25)	43/162 (27)	11/54 (20)	0.4
Complete response (pCR)	36/216 (17)	29/162 (18)	7/54 (13)	0.4
Any response (no pOR or pCR)	103/216 (48)	68/162 (42)	35/54 (65)	0.004
No response	59/216 (27)	51/162 (31)	8/54 (15)	0.017
Urinary diversion, *n*/*N* (%)				0.7
Ileal conduit	145/193 (75)	105/141 (74)	40/52 (77)	
Neobladder	44/193 (23)	32/141 (23)	12/52 (23)	
Pouch	4/193 (2.1)	4/141 (2.8)	0/52 (0)	
Extent of LND, *n*/*N* (%)				0.3
Standard	74/150 (49)	58/114 (51)	16/36 (44)	
Extended	70/150 (47)	50/114 (44)	20/36 (56)	
Super-extended	6/150 (4.0)	6/114 (5.3)	0/36 (0)	
Mean LNs removed, *n* (SD)	19.6 (12.5)	20.1 (12.9)	18.4 (11.2)	0.5
Data missing	15	14	1	
Mean positive LNs, *n* (SD)	2.0 (3.5)	2.0 (3.8)	1.9 (2.8)	0.6
Data missing	9	9	0	
Positive surgical margins, *n*/*N* (%)	26/216 (12)	17/162 (10)	9/54 (17)	0.2
Concomitant CIS at RC, *n*/*N* (%)	48/214 (22)	32/160 (20)	16/54 (30)	0.14

CIS = carcinoma in situ; LN = lymph nodes; LND = LN dissection; RC = radical cystectomy; SD = standard deviation; TURBT = transurethral resection of bladder tumor.

a Percentages may not exactly add up to 100% as they are rounded.

b Wilcoxon rank-sum test, χ2 test, or Fisher’s exact test, as appropriate.

### 3.2 Pathological outcomes

Overall, 25% of the patients had pOR and 17% pCR at RC. In the cisplatin group, 43 patients (27%) had pOR and 29 (18%) had pCR. In the carboplatin group, 11 patients (20%) had pOR and seven (13%) had pCR. A pathological nodal status of ypN0 was observed for 77 patients (48%) treated with cisplatin-based IC and 25 patients (46%) treated with gemcitabine/carboplatin. Table 2 summarizes ypN distribution by cN status. Multivariable logistic regression analyses did not identify independent predictors of pOR or pCR. cN2 status was a predictor of ypN status at RC (*p* = 0.03; Supplementary Table 2). In the cN1 subgroup, 27 patients (24%) experienced pOR and 16 (14%) experienced pCR at RC. In the cN1 subgroup, there was no significant difference in the pOR rate (22% vs 30%) or pCR rate (13% vs 19%) between the IC regimens (both *p* > 0.3).

**Table 2:** Pathological N status distribution by clinical N status in a propensity score–matched cohort of 216 patients treated with induction chemotherapy and radical cystectomy with lymphadenectomy for cT2–4 N1–3 M0 bladder cancer a

Clinical N stage	Pathological N stage, *n* (%)				
	ypN0	ypN1	ypN2	ypN3	Total
cN1	59 (27)	24 (11)	25 (12)	6 (2.8)	114 (53)
cN2	37 (17)	15 (6.9)	25 (12)	11 (5.1)	88 (41)
cN3	6 (2.8)	2 (0.9)	3 (1.4)	3 (1.4)	14 (6.5)
Total	102 (47)	41 (19)	53 (25)	20 (9.3)	216 (100)

a Percentages may not exactly add up to 100% as they are rounded.

### 3.3 Survival analysis

Median follow-up after RC for patients still alive was 23.4 mo (interquartile range 10.3–45.4). Overall, 110 patients (50.9%) developed recurrence. In total, 107 patients (49.5%) died, with 93 (43.1%) dying of BCa. In the cisplatin group, the estimated median OS was 37 mo (95% confidence interval [CI] 27.4–78) and median CSS was 39.2 mo (95% CI 28–not reached). In the carboplatin group, the estimated median OS was 15 mo (95% CI 10–24.8) and median CSS was 16 mo (95% CI 11–not reached).

**Fig. 1:**
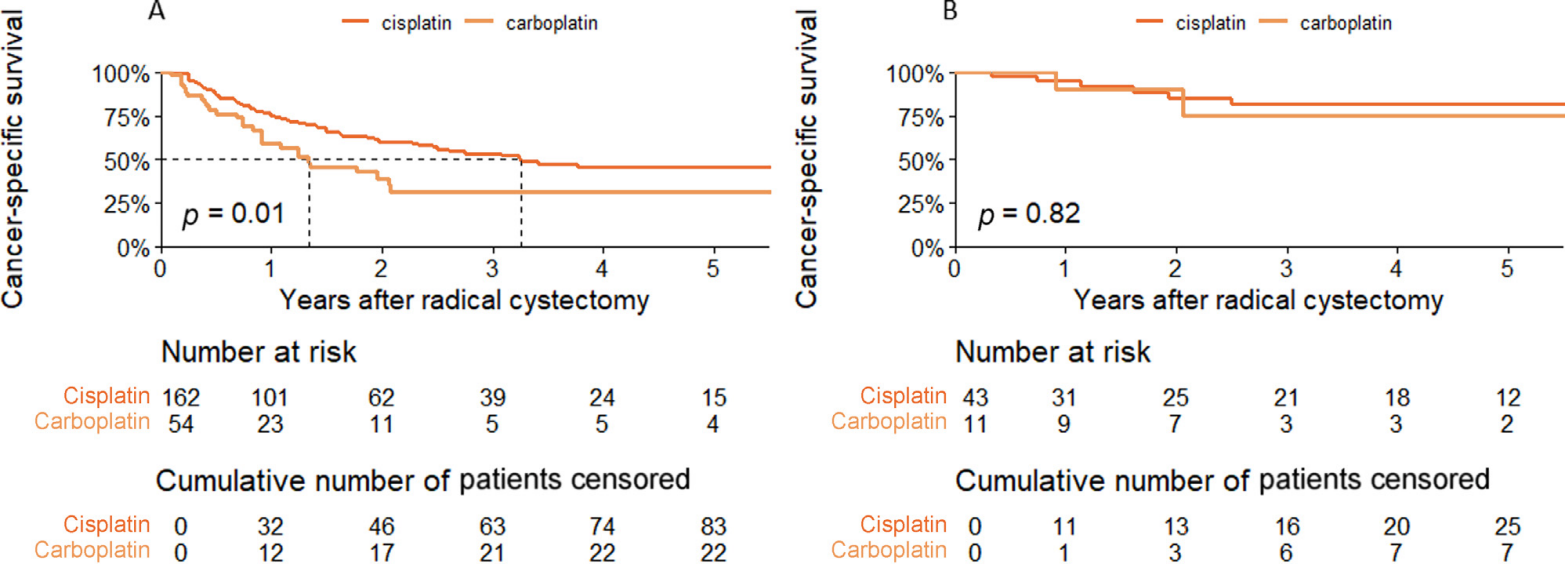
Kaplan-Meier cancer-specific survival plots for (A) all 216 propensity score–matched patients and (B) all 54 propensity score–matched patients with a pathological complete or partial response. All patients were treated with induction chemotherapy and radical cystectomy with lymphadenectomy for cT2–4 N1–3 M0 bladder cancer. Results are stratified by chemotherapy regimen.

The 2-yr OS and CSS estimates were 58.1% (95% CI 50.2–67.2%) and 59.8% (95% CI 51.9–69) in the cisplatin group, compared to 34.1% (95% CI 22.3–52.2) and 38.8% (95% CI 26–57.9) in the carboplatin group, respectively (CSS is shown in Fig. 1A). Figure 1B shows Kaplan-Meier CSS curves for patients with pOR; there was no significant difference in survival between the IC regimens.

**Fig. 2:**
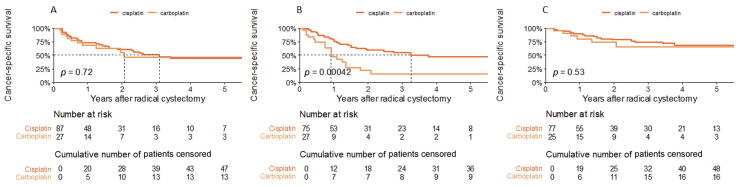
Kaplan-Meier cancer-specific survival plots for (A) all 114 patients with cN1 status, (B) all 102 patients with cN2/3 status, and (C) all 102 patients with ypN0 status from the cohort of 216 propensity score–matched patients treated with induction chemotherapy and radical cystectomy with lymphadenectomy for cT2–4 N1–3 M0 bladder cancer. Results are stratified by chemotherapy regimen.

For the cN1 subgroup, there was no significant difference in OS (*p* = 0.77) or CSS (*p* = 0.7; Fig. 2A) between the IC regimens, but there was a difference in CSS in favor of cisplatin in the cN2/3 subgroup (*p* < 0.001; Fig. 2B). In the ypN0 subgroup, there was no significant difference in CSS (*p* = 0.5; Fig. 2C) or OS (*p* = 0.07) between the IC regimens.

On univariable Cox regression analysis, treatment with gemcitabine/carboplatin was significantly associated with shorter OS (hazard ratio [HR] 1.89, 95% CI 1.25––2.85; *p* = 0.002) and shorter CSS (HR 1.78, 95% CI 1.14–2.78; *p* = 0.011). On multivariable Cox regression analyses adjusted for the effects of potential confounders, treatment with gemcitabine/carboplatin (*p* < 0.001, *p* = 0.003), ypT3 (*p* = 0.007, *p* = 0.003), ypT4 (*p* = 0.04, *p* = 0.014), ypN2 (*p* < 0.001, *p* < 0.001), ypN3 (*p* = 0.003, *p* < 0.001), and positive surgical margins (*p* < 0.001, *p* < 0.001) were all significantly associated with shorter OS and shorter CSS (Table 3). The models showed good discrimination ability (C index 0.79 for OS and 0.82 for CSS).

**Table 3:** Multivariable Cox regression analysis for prognostication of cancer-specific survival and overall survival in a propensity score–matched cohort of 216 patients treated with induction chemotherapy and radical cystectomy with lymphadenectomy for cT2–4 N1–3 M0 bladder cancer

	Cancer-specific survival		Overall survival	
	HR (95% CI)	*p* value	HR (95% CI)	*p* value
Carboplatin regimen (vs cisplatin)	2.81 (1.43–5.52)	**0.003**	2.86 (1.56–5.25)	**<0.001**
Age	0.99 (0.95–1.02)	0.4	0.99 (0.96–1.03)	0.7
Male sex (vs female)	0.64 (0.34–1.20)	0.2	0.77 (0.42–1.40)	0.4
Smoking status (vs no smoking)	1.08 (0.62–1.87)	0.8	1.15 (0.69–1.94)	0.6
Number of cycles (vs ≤3 cycles)				
4 cycles	2.27 (1.00–5.17)	0.051	1.98 (0.92–4.25)	0.080
≥5 cycles	1.82 (0.71–4.69)	0.2	1.57 (0.65–3.77)	0.3
Pathological T stage (vs ypT0)				
ypTa/pTis/pT1	0.95 (0.23–3.87)	>0.9	0.73 (0.22–2.47)	0.6
ypT2	0.73 (0.15–3.61)	0.7	1.48 (0.48–4.54)	0.5
ypT3	3.44 (1.51–7.84)	**0.003**	2.77 (1.33–5.76)	**0.007**
ypT4	3.36 (1.27–8.84)	**0.014**	2.53 (1.04–6.13)	**0.040**
Pathological N stage (vs ypN0)				
ypN1	1.15 (0.47–2.79)	0.8	0.92 (0.41–2.06)	0.8
ypN2	4.23 (2.04–8.73)	**<0.001**	3.65 (1.87–7.14)	**<0.001**
ypN3	4.03 (1.77–9.16)	**<0.001**	3.33 (1.52–7.27)	**0.003**
Positive surgical margins (vs negative)	5.48 (2.33–12.9)	**<0.001**	5.35 (2.45–11.7)	**<0.001**
≥16 LNs removed (vs ≤15)	0.94 (0.53–1.66)	0.8	0.86 (0.51–1.46)	0.6
Concomitant CIS at RC (vs no CIS)	1.02 (0.49–2.14)	>0.9	0.93 (0.46–1.86)	0.8
C index	0.82		0.79	

CIS = carcinoma in situ; CI = confidence interval; HR = hazard ratio; LNs = lymph nodes; RC = radical cystectomy.

On subgroup analyses, there was no association between gemcitabine/carboplatin and survival outcomes in the cN1 subgroup (*p* = 0.2, *p* = 0.1), whereas in the cN2/3 subgroup treatment with gemcitabine/carboplatin was associated with shorter OS (HR 4.84, 95% CI 2.13–11.0; *p* < 0.001) and shorter CSS (HR 4.48, 95% CI 1.81–11.1; *p* = 0.001; Supplementary Tables 3 and 4).

## 4 Discussion

Oncological outcomes of cisplatin-based chemotherapy are considered superior to those with gemcitabine/carboplatin for cisplatin-eligible patients with BCa. In our study, the pOR rate was 27% for platinum-based IC among patients with cN+ BCa, and the pOR rate with cisplatin-based combination IC was 7% better than with gemcitabine/carboplatin, although the difference was not statistically significant (*p* = 0.4). Cisplatin-based combination IC conferred significant OS and CSS benefits in comparison to gemcitabine/carboplatin IC, driven mainly by the benefit for patients with cN2–N3 disease. These results align with previous small phase 2 studies that showed favorable response rates [19] and median disease-related survival [20] for cisplatin-based chemotherapy in comparison to carboplatin-based regimens. However, the only phase 3 head-to-head comparison of cisplatin-based versus carboplatin-based combination chemotherapy in cisplatin-eligible patients did not find a significant difference in median OS [21]. Moreover, on the basis of randomized controlled trials in the immunotherapy era (DANUBE, IMvigor130, KEYNOTE-361), it was suggested that carboplatin treatment in metastatic BCa may be more effective than previously thought. A recent network meta-analysis of these trials demonstrated that cisplatin- and carboplatin-based chemotherapy had similar efficacy in terms of OS and pCR rates [22].

In the cN1 subgroup, there was no significant difference in OS, CSS, or pOR between the cisplatin and gemcitabine/carboplatin IC groups. Of the cN1 subgroup, 24% experienced a pOR, highlighting the real benefit of IC followed by RC in patients with limited nodal involvement. This may reflect the more beneficial nature of IC in cN1 in comparison to cN2–3 stages [12]. Furthermore, patients with cN1 disease are more likely to have false-positive imaging in comparison to patients with cN2/3 disease [11]. However, patients with cN+ status that remain ypN-positive after RC have significantly worse survival outcomes, regardless of cN stage [12], [13], [23]. Moreover, a multi-institutional analysis of 304 patients with cN+ BCa treated with IC and RC did not find a significant difference in OS between cN1 and cN2–3 cases [13]. In addition, more than 45% of the patients with cN1 stage in our study remained ypN-positive at final pathology. These findings support the overall value of IC and the potential benefit of gemcitabine/carboplatin in patients with cN1 BCa who are ineligible for cisplatin.

Several factors support the use of carboplatin-based combination chemotherapy in cisplatin-ineligible patients with cN+ BCa instead of surgery alone. While approximately 25% of patients with ypN-positive disease can be cured with RC and extended PLND alone [24], a population-based study showed that 5-yr OS in cN+ BCa was 31% for patients treated with preoperative chemotherapy followed by RC versus 19% for patients who underwent RC alone [14]. For cisplatin-ineligible patients with advanced urothelial cancer, depending on the regimen (methotrexate/carboplatin/vinblastine vs gemcitabine/carboplatin), carboplatin induced overall response rates that ranged between 30.3% and 41.2% [16]. However, the sequence for carboplatin administration may be important. A retrospective study recently showed that ypN-positive patients at RC did not benefit from carboplatin-based adjuvant chemotherapy when compared to RC alone [25]. Of note, none of these patients had received preoperative chemotherapy and the study did not provide data on clinical node stage [25]. In summary, chemotherapy or RC alone are generally insufficient to cure metastatic BCa regardless of the regimen, suggesting an overall benefit from a multimodal treatment approach in the cN+ setting, even in cisplatin-ineligible patients [14], [26], [27], [28].

We acknowledge that our study has several limitations. First, owing to the retrospective design, the small sample size, and the limited number of events in the carboplatin group, the study may have been underpowered for detection of a difference between the two treatment groups. In addition, despite no significant differences in baseline variables between the groups after PSM, our cohort may be subject to an inherent selection bias. Nonetheless, the data provide evidence for further prospective hypothesis testing. Second, data on renal function and on cisplatin ineligibility would be helpful for better stratification of patients in addition to the quality criteria applied and better correction for cofounders during statistical analyses. Moreover, we do not know whether cisplatin-eligible patients received gemcitabine/carboplatin and we do not report on further cisplatin ineligibility criteria, including performance scores, regimen switches, the treatment dose, or IC-induced toxicities. Third, we do not provide data on restaging after IC to assess the clinical response to IC and did not include patients who experienced progression on IC and therefore did not undergo RC. This may pose a bias towards patients with a better response to IC and a lower progression rate in one of the treatment groups. In general, RC was only performed if there was no obvious progression during IC. Fourth, we acknowledge the limitations concerning the accuracy of clinical staging and the benefits of modern staging technologies [10]. Nonetheless, more than 50% of the patients remained ypN-positive at RC, indicating correct clinical prediction of lymph node metastasis in these cases. Moreover, RC with PLND is the gold standard for lymph node staging. Fifth, the multicenter design means that multiple surgeons were involved and there was no standardized IC schedule or standardized staging or assessment of pathological specimens, and several cisplatin-based regimens were considered. We tried to mitigate these shortcomings by applying PSM.

In summary, if patients respond to IC, gemcitabine/carboplatin results in similar survival outcomes to those with cisplatin-based regimens. Furthermore, there was no survival difference among cN1 patients when stratified by IC regimen. Thus, carboplatin should be a treatment option for selected cisplatin-ineligible patients with BCa who may benefit from intensification of gemcitabine/carboplatin with novel therapies or novel targeted therapies combining immune therapies and possibly enfortumab vedotin (eg, NCT05239624) [29], [30].

## 5 Conclusions

Cisplatin-based IC seems to be superior to gemcitabine/carboplatin and should be the standard for patients with cisplatin-eligible cN+ BCa. Nevertheless, carboplatin-based IC treatment appears to be an attractive alternative for patients with cisplatin-ineligible cN+ BCa before RC. In particular, selected cisplatin-ineligible patients with cN1 disease may benefit from gemcitabine/carboplatin IC. Patients who experienced a pathological response to IC had better survival regardless of the IC regimen. cN+ BCa is a heterogeneous disease that deserves better risk stratification to guide clinical decision-making.

  ***Author contributions***: Shahrokh F. Shariat had full access to all the data in the study and takes responsibility for the integrity of the data and the accuracy of the data analysis.

  *Study concept and design*: von Deimling, Shariat, Pradere.

*Acquisition of data*: von Deimling, Mertens, van Rhijn, Lotan, Spiess, Daneshmand, Black, D’Andrea, Moschini, Soria, Afferi, Roumiguié, Pradere.

*Analysis and interpretation of data*: von Deimling, Shariat, Pradere.

*Drafting of the manuscript*: von Deimling, Shariat, Pradere.

*Critical revision of the manuscript for important intellectual content*: Mertens, van Rhijn, Lotan, Spiess, Daneshmand, Black, Pallauf, D’Andrea, Moschini, Soria, Del Giudice, Afferi, Laukhtina, Yanagisawa, Kawada, Teoh, Abufaraj, Ploussard, Roumiguié, Karakiewicz, Babjuk, Gontero, Xylinas, Rink, Shariat, Pradere.

*Statistical analysis*: von Deimling.

*Obtaining funding*: None.

*Administrative, technical, or material support*: None.

*Supervision*: Shariat, Pradere.

*Other*: None.

  ***Financial disclosures:*** Shahrokh F. Shariat certifies that all conflicts of interest, including specific financial interests and relationships and affiliations relevant to the subject matter or materials discussed in the manuscript (eg, employment/affiliation, grants or funding, consultancies, honoraria, stock ownership or options, expert testimony, royalties, or patents filed, received, or pending), are the following: Philippe E. Spiess serves as a vice-chair of the National Comprehensive Cancer Network guidelines panel for bladder and penile cancer. Maximilian Pallauf has received a research grant from the Austrian Urological Association, support for attending the Austrian Urological Association yearly meeting, speaker honoraria from Astellas, Janssen, and MedMedia, and an honorarium from Spectra for attending an advisory board; he was a board member for the Austrian Urological Association from 2018 to 2021. Shahrokh F. Shariat has received honoraria from Astellas, AstraZeneca, BMS, Ferring, Ipsen, Janssen, MSD, Olympus, Pfizer, Roche, and Takeda; has a consulting or advisory role for Astellas, AstraZeneca, BMS, Ferring, Ipsen, Janssen, MSD, Olympus, Pfizer, Pierre Fabre, Roche, and Takeda; and participates in speaker bureaus for Astellas, AstraZeneca, Bayer, BMS, Ferring, Ipsen, Janssen, MSD, Olympus, Pfizer, Richard Wolf, Roche, and Takeda. The remaining authors have nothing to disclose.

  ***Funding/Support and role of the sponsor*:** None.
