# Supplementary material for: Carboplatin Induction Chemotherapy in Clinically Lymph Node–positive Bladder Cancer
Source: Eur Urol Open Sci. 2023 Mar 25;51:39–46. doi: 10.1016/j.euros.2023.02.014 (PMC10175724; doi:10.1016/j.euros.2023.02.014)

**Supplementary Figure 1:** Standardized mean differences prior to and after propensity-score matching in 369 and 216 patients treated with induction chemotherapy and radical cystectomy with lymphadenectomy for cT2-4N1-3M0 bladder cancer. Matching was performed using age, gender, presence of carcinoma in situ (CIS) at transurethral resection of bladder tumor (TURBT), clinical tumor, and clinical node stage.


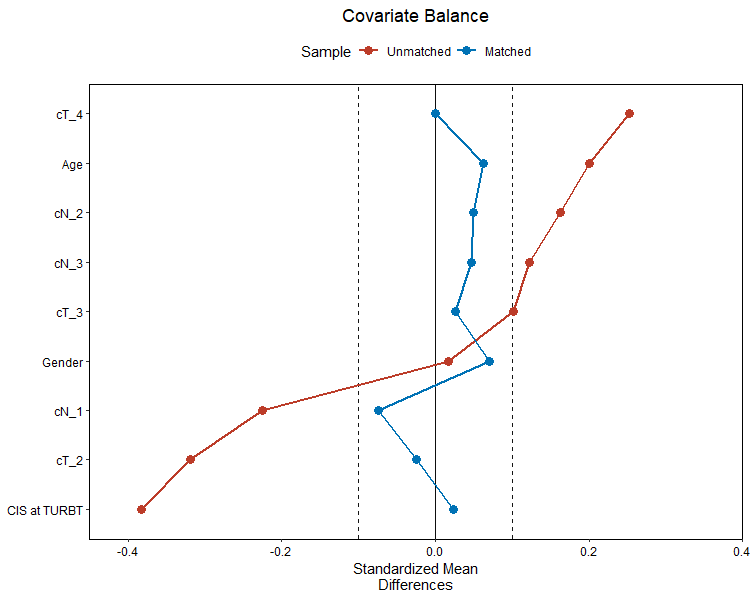

Supplement: Supplementary data 1 [file mmc1.docx]
