# Supplementary material for: Carboplatin Induction Chemotherapy in Clinically Lymph Node–positive Bladder Cancer
Source: Eur Urol Open Sci. 2023 Mar 25;51:39–46. doi: 10.1016/j.euros.2023.02.014 (PMC10175724; doi:10.1016/j.euros.2023.02.014)
Supplement: Supplementary data 2 [file mmc2.docx]

| **Supplementary Table 1: Baseline characteristics of 369 patients prior to propensity-score matching treated with induction chemotherapy and radical cystectomy with lymphadenectomy for cT2-4N1-3M0 bladder cancer.** | | | | |
| --- | --- | --- | --- | --- |
|  | **Overall** | **Regimen** | | |
|  | **N = 369** | **Cisplatin**  **N = 315** | **Carboplatin**  **N = 54** | **p-value** |
| **Age** | 64.5 (9.1) | 64.2 (9.0) | 66.2 (9.8) | 0.2 |
| Unknown | 1 | 1 | 0 |  |
| **Sex** |  |  |  | 0.9 |
| Female | 93 / 369 (25%) | 79 / 315 (25%) | 14 / 54 (26%) |  |
| Male | 276 / 369 (75%) | 236 / 315 (75%) | 40 / 54 (74%) |  |
| **Smoking history** |  |  |  | 0.10 |
| Current smoker | 77 / 287 (27%) | 71 / 250 (28%) | 6 / 37 (16%) |  |
| Never smoker | 91 / 287 (32%) | 74 / 250 (30%) | 17 / 37 (46%) |  |
| Past smoker | 119 / 287 (41%) | 105 / 250 (42%) | 14 / 37 (38%) |  |
| **CIS at TURBT** | 58 / 365 (16%) | 54 / 311 (17%) | 4 / 54 (7.4%) | 0.065 |
| **Clinical T stage** |  |  |  | **0.046** |
| cT2 | 208 / 369 (56%) | 185 / 315 (59%) | 23 / 54 (43%) |  |
| cT3 | 101 / 369 (27%) | 84 / 315 (27%) | 17 / 54 (31%) |  |
| cT4 | 60 / 369 (16%) | 46 / 315 (15%) | 14 / 54 (26%) |  |
| **Clinical N stage** |  |  |  | 0.2 |
| cN1 | 221 / 369 (60%) | 194 / 315 (62%) | 27 / 54 (50%) |  |
| cN2 | 131 / 369 (36%) | 108 / 315 (34%) | 23 / 54 (43%) |  |
| cN3 | 17 / 369 (4.6%) | 13 / 315 (4.1%) | 4 / 54 (7.4%) |  |
| **Variant histology at TURBT** | 35 / 369 (9.5%) | 30 / 315 (9.5%) | 5 / 54 (9.3%) | >0.9 |
| **Number of cycles** |  |  |  | 0.6 |
| ≤ 3 cycles | 102 / 369 (28%) | 86 / 315 (27%) | 16 / 54 (30%) |  |
| 4 cycles | 194 / 369 (53%) | 169 / 315 (54%) | 25 / 54 (46%) |  |
| ≥ 5 cycles | 73 / 369 (20%) | 60 / 315 (19%) | 13 / 54 (24%) |  |
| **Pathological T stage** |  |  |  | 0.2 |
| ypT0 | 90 / 369 (24%) | 77 / 315 (24%) | 13 / 54 (24%) |  |
| ypTa/pTis/pT1 | 56 / 369 (15%) | 48 / 315 (15%) | 8 / 54 (15%) |  |
| ypT2 | 56 / 369 (15%) | 53 / 315 (17%) | 3 / 54 (5.6%) |  |
| ypT3 | 107 / 369 (29%) | 90 / 315 (29%) | 17 / 54 (31%) |  |
| ypT4 | 60 / 369 (16%) | 47 / 315 (15%) | 13 / 54 (24%) |  |
| **Pathological N stage** |  |  |  | 0.6 |
| ypN0 | 179 / 369 (49%) | 154 / 315 (49%) | 25 / 54 (46%) |  |
| ypN1 | 80 / 369 (22%) | 68 / 315 (22%) | 12 / 54 (22%) |  |
| ypN2 | 78 / 369 (21%) | 68 / 315 (22%) | 10 / 54 (19%) |  |
| ypN3 | 32 / 369 (8.7%) | 25 / 315 (7.9%) | 7 / 54 (13%) |  |
| **Pathological N status** |  |  |  | 0.7 |
| ypN+ | 190 / 369 (51%) | 161 / 315 (51%) | 29 / 54 (54%) |  |
| **Objective Response (pOR)** | 144 / 369 (39%) | 127 / 315 (40%) | 17 / 54 (31%) | 0.2 |
| **Complete Response (pCR)** | 62 / 369 (17%) | 55 / 315 (17%) | 7 / 54 (13%) | 0.4 |
| **Any Response (no pOR or pCR)** | 121 / 369 (33%) | 92 / 315 (29%) | 29 / 54 (54%) | **<0.001** |
| **No Response** | 104 / 369 (28%) | 96 / 315 (30%) | 8 / 54 (15%) | **0.018** |
| **Urinary diversion** |  |  |  | 0.3 |
| Ileal conduit | 221 / 328 (67%) | 181 / 276 (66%) | 40 / 52 (77%) |  |
| Neobladder | 92 / 328 (28%) | 80 / 276 (29%) | 12 / 52 (23%) |  |
| Pouch | 14 / 328 (4.3%) | 14 / 276 (5.1%) | 0 / 52 (0%) |  |
| Ureterocutaneostomy | 1 / 328 (0.3%) | 1 / 276 (0.4%) | 0 / 52 (0%) |  |
| **Extent of LND** |  |  |  | 0.5 |
| Standard | 116 / 256 (45%) | 100 / 220 (45%) | 16 / 36 (44%) |  |
| Extended | 129 / 256 (50%) | 109 / 220 (50%) | 20 / 36 (56%) |  |
| Super extended | 11 / 256 (4.3%) | 11 / 220 (5.0%) | 0 / 36 (0%) |  |
| **Number of LN removed** | 19.8 (12.3) | 20.1 (12.5) | 18.4 (11.2) | 0.4 |
| Unknown | 32 | 31 | 1 |  |
| **Number of positive LN** | 1.9 (3.8) | 1.9 (3.9) | 1.9 (2.8) | 0.3 |
| Unknown | 20 | 20 | 0 |  |
| **Positive surgical margins** | 44 / 369 (12%) | 35 / 315 (11%) | 9 / 54 (17%) | 0.2 |
| **Concomitant CIS at RC** | 101 / 365 (28%) | 85 / 311 (27%) | 16 / 54 (30%) | 0.7 |
| CIS = Carcinoma in situ, LN = Lymph Nodes, LND = Lymph Node Dissection, RC = Radical Cystectomy, TURBT = Transurethral Resection Of Bladder Tumor | | | | |
| Mean (SD); n / N (%); Wilcoxon rank sum test; Chi-squared test; Fisher's exact test | | | | |
| Percentages may not exactly add up to 100%, as they are rounded. | | | | |
