# Supplementary material for: Carboplatin Induction Chemotherapy in Clinically Lymph Node–positive Bladder Cancer
Source: Eur Urol Open Sci. 2023 Mar 25;51:39–46. doi: 10.1016/j.euros.2023.02.014 (PMC10175724; doi:10.1016/j.euros.2023.02.014)
Supplement: Supplementary data 3 [file mmc3.docx]

| **Supplementary Table 2: Multivariable logistic regression analysis predicting the pathological response to induction chemotherapy (IC) in 216 propensity-score matched patients treated with IC and radical cystectomy with lymphadenectomy for cT2-4N1-3M0 bladder cancer.** | | | | | | | | | | | | |
| --- | --- | --- | --- | --- | --- | --- | --- | --- | --- | --- | --- | --- |
|  | **Complete Response** | | | **Objective Response** | | | **No Response** | | | **Pathological N-status** | | |
|  | **OR** | **95% CI** | **p-value** | **OR** | **95% CI** | **p-value** | **OR** | **95% CI** | **p-value** | **OR** | **95% CI** | **p-value** |
| **Regimen (ref: cisplatin)** |  |  |  |  |  |  |  |  |  |  |  |  |
| Carboplatin | 0.47 | 0.13, 1.34 | 0.2 | 0.54 | 0.20, 1.31 | 0.2 | 0.36 | 0.10, 1.03 | 0.080 | 1.33 | 0.62, 2.89 | 0.5 |
| **Smoking status (ref: no)** | 0.93 | 0.41, 2.17 | 0.9 | 0.63 | 0.31, 1.28 | 0.2 | 0.76 | 0.34, 1.73 | 0.5 | 0.83 | 0.43, 1.58 | 0.6 |
| **Number of cycles (ref: ≤ 3)** |  |  |  |  |  |  |  |  |  |  |  |  |
| 4 cycles | 2.11 | 0.70, 7.90 | 0.2 | 1.75 | 0.69, 4.87 | 0.3 | 1.19 | 0.46, 3.23 | 0.7 | 0.84 | 0.36, 1.92 | 0.7 |
| ≥ 5 cycles | 1.52 | 0.39, 6.61 | 0.6 | 1.40 | 0.46, 4.49 | 0.6 | 0.76 | 0.22, 2.54 | 0.7 | 0.94 | 0.36, 2.47 | 0.9 |
| **Clinical T stage (ref: cT2)** |  |  |  |  |  |  |  |  |  |  |  |  |
| cT3 | 1.09 | 0.38, 3.17 | 0.9 | 0.94 | 0.38, 2.30 | 0.9 | 1.72 | 0.70, 4.32 | 0.2 | 1.62 | 0.74, 3.55 | 0.2 |
| cT4 | 1.67 | 0.63, 4.58 | 0.3 | 1.43 | 0.61, 3.33 | 0.4 | 0.29 | 0.07, 0.89 | 0.043 | 1.05 | 0.48, 2.31 | 0.9 |
| **Clinical N stage (ref: cN1)** |  |  |  |  |  |  |  |  |  |  |  |  |
| cN2 | 1.53 | 0.64, 3.78 | 0.3 | 0.99 | 0.46, 2.12 | >0.9 | 0.68 | 0.28, 1.59 | 0.4 | 2.15 | 1.09, 4.29 | **0.029** |
| cN3 | 0.41 | 0.02, 2.51 | 0.4 | 0.70 | 0.14, 2.70 | 0.6 | 0.55 | 0.07, 2.60 | 0.5 | 1.88 | 0.54, 6.64 | 0.3 |
| CI = Confidence Interval, OR = Odds Ratio | | | | | | | | | | | | |
