# Supplementary material for: Carboplatin Induction Chemotherapy in Clinically Lymph Node–positive Bladder Cancer
Source: Eur Urol Open Sci. 2023 Mar 25;51:39–46. doi: 10.1016/j.euros.2023.02.014 (PMC10175724; doi:10.1016/j.euros.2023.02.014)
Supplement: Supplementary data 4 [file mmc4.docx]

| **Supplementary Table 3: Multivariable Cox regression analysis for prognostication of cancer-specific survival and overall survival in 114 propensity-score matched patients treated with induction chemotherapy and radical cystectomy with lymphadenectomy for cT2-4N1M0 bladder cancer.** | | | | | | |
| --- | --- | --- | --- | --- | --- | --- |
|  | **Cancer-specific survival** | | | **Overall survival** | | |
|  | **HR** | **95% CI** | **p-value** | **HR** | **95% CI** | **p-value** |
| **Regimen (ref: cisplatin)** |  |  |  |  |  |  |
| Carboplatin | 3.55 | 0.79, 15.9 | 0.1 | 2.45 | 0.68, 8.86 | 0.2 |
| **Age** | 1.00 | 0.92, 1.08 | >0.9 | 1.01 | 0.94, 1.09 | 0.7 |
| **Sex (ref: female)** |  |  |  |  |  |  |
| Male | 0.49 | 0.13, 1.80 | 0.3 | 0.71 | 0.22, 2.28 | 0.6 |
| **Smoking status (ref: no)** | 1.94 | 0.53, 7.11 | 0.3 | 1.81 | 0.57, 5.67 | 0.3 |
| **Number of cycles (ref: ≤ 3 cycles)** |  |  |  |  |  |  |
| 4 cycles | 3.65 | 0.65, 20.3 | 0.14 | 2.82 | 0.68, 11.7 | 0.2 |
| ≥ 5 cycles | 3.03 | 0.32, 28.5 | 0.3 | 1.62 | 0.23, 11.3 | 0.6 |
| **Pathological T stage (ref: ypT0)** |  |  |  |  |  |  |
| ypTa/pTis/pT1 | 0.93 | 0.06, 15.3 | >0.9 | 0.94 | 0.12, 7.38 | >0.9 |
| ypT2 | 0.44 | 0.03, 5.55 | 0.5 | 0.78 | 0.12, 4.92 | 0.8 |
| ypT3 | 2.59 | 0.41, 16.3 | 0.3 | 1.80 | 0.40, 8.15 | 0.4 |
| ypT4 | 1.72 | 0.19, 15.9 | 0.6 | 1.27 | 0.20, 7.89 | 0.8 |
| **Pathological N stage (ref: ypN0)** |  |  |  |  |  |  |
| ypN1 | 1.94 | 0.29, 13.0 | 0.5 | 2.11 | 0.41, 10.9 | 0.4 |
| ypN2 | 8.83 | 1.88, 41.5 | **0.006** | 6.46 | 1.59, 26.2 | **0.009** |
| ypN3 | 24.4 | 2.45, 243 | **0.006** | 21.2 | 2.20, 203 | **0.008** |
| **Positive surgical margins (ref: no)** | 10.3 | 1.77, 60.0 | **0.009** | 12.5 | 2.69, 58.4 | **0.001** |
| **Number of LN removed (ref: ≤ 15)** |  |  |  |  |  |  |
| ≥ 16 | 0.71 | 0.24, 2.11 | 0.5 | 0.93 | 0.37, 2.31 | 0.9 |
| **Concomitant CIS at RC (ref: no)** | 1.46 | 0.34, 6.19 | 0.6 | 0.79 | 0.21, 3.06 | 0.7 |
| **C-Index** |  | | 0.86 |  | | 0.81 |
| CIS = Carcinoma in situ, CI = Confidence Interval, HR = Hazard Ratio, LN = Lymph Nodes, RC = Radical Cystectomy | | | | | | |
