# Supplementary material for: Carboplatin Induction Chemotherapy in Clinically Lymph Node–positive Bladder Cancer
Source: Eur Urol Open Sci. 2023 Mar 25;51:39–46. doi: 10.1016/j.euros.2023.02.014 (PMC10175724; doi:10.1016/j.euros.2023.02.014)
Supplement: Supplementary data 5 [file mmc5.docx]

| **Supplementary Table 4: Multivariable Cox regression analysis for prognostication of cancer-specific survival and overall survival in 102 propensity-score matched patients treated with induction chemotherapy and radical cystectomy with lymphadenectomy for cT2-4N2-3M0 bladder cancer.** | | | | | | |
| --- | --- | --- | --- | --- | --- | --- |
|  | **Cancer-specific survival** | | | **Overall survival** | | |
|  | **HR** | **95% CI** | **p-value** | **HR** | **95% CI** | **p-value** |
| **Regimen (ref: cisplatin)** |  |  |  |  |  |  |
| Carboplatin | 4.48 | 1.81, 11.1 | **0.001** | 4.84 | 2.13, 11.0 | **<0.001** |
| **Age** | 0.97 | 0.93, 1.02 | 0.3 | 0.98 | 0.94, 1.03 | 0.5 |
| **Sex (ref: female)** |  |  |  |  |  |  |
| Male | 1.08 | 0.43, 2.74 | 0.9 | 1.29 | 0.52, 3.20 | 0.6 |
| **Smoking status (ref: no)** | 0.75 | 0.39, 1.45 | 0.4 | 0.87 | 0.46, 1.65 | 0.7 |
| **Number of cycles (ref: ≤ 3 cycles)** |  |  |  |  |  |  |
| 4 cycles | 1.91 | 0.59, 6.18 | 0.3 | 1.86 | 0.61, 5.67 | 0.3 |
| ≥ 5 cycles | 1.32 | 0.37, 4.74 | 0.7 | 1.41 | 0.42, 4.76 | 0.6 |
| **Pathological T stage (ref: ypT0)** |  |  |  |  |  |  |
| ypTa/pTis/pT1 | 1.35 | 0.22, 8.36 | 0.7 | 0.71 | 0.13, 3.96 | 0.7 |
| ypT2 | 1.27 | 0.12, 13.5 | 0.8 | 2.53 | 0.49, 13.0 | 0.3 |
| ypT3 | 4.69 | 1.56, 14.1 | **0.006** | 3.51 | 1.31, 9.43 | **0.013** |
| ypT4 | 8.12 | 2.01, 32.7 | **0.003** | 5.19 | 1.45, 18.6 | **0.011** |
| **Pathological N stage (ref: ypN0)** |  |  |  |  |  |  |
| ypN1 | 1.34 | 0.36, 4.95 | 0.7 | 0.93 | 0.29, 3.02 | >0.9 |
| ypN2 | 5.42 | 1.91, 15.4 | **0.001** | 3.57 | 1.45, 8.80 | **0.006** |
| ypN3 | 3.30 | 1.15, 9.46 | **0.026** | 2.25 | 0.87, 5.83 | 0.10 |
| **Positive surgical margins (ref: no)** | 4.49 | 1.47, 13.8 | **0.008** | 3.50 | 1.27, 9.65 | **0.015** |
| **Number of LN removed (ref: ≤ 15)** |  |  |  |  |  |  |
| ≥ 16 | 1.27 | 0.54, 2.99 | 0.6 | 0.83 | 0.36, 1.92 | 0.7 |
| **Concomitant CIS at RC (ref: no)** | 0.68 | 0.24, 1.93 | 0.5 | 0.67 | 0.25, 1.81 | 0.4 |
| **C-Index** |  | | 0.81 |  | | 0.78 |
| CIS = Carcinoma in situ, CI = Confidence Interval, HR = Hazard Ratio, LN = Lymph Nodes, RC = Radical Cystectomy | | | | | | |
